# “All the horrible emotions have passed, I still remained, and I was safe”: A qualitative study of Lesbian and Gay people's lived experience of completing a full Dialectical Behaviour Therapy programme

**DOI:** 10.1111/papt.12555

**Published:** 2024-10-30

**Authors:** Charlotte Harding, Daniel Pratt, James Lea

**Affiliations:** ^1^ Division of Psychology and Mental Health, Faculty of Biology, Medicine, and Health, School of Social Sciences University of Manchester Manchester UK; ^2^ Greater Manchester Mental Health NHS Foundation Trust Manchester UK; ^3^ Greater Manchester Mental Health NHS Foundation Trust, Suicide, Risk and Safety Research Unit Manchester Academic Health Science Centre (MAHSC) Manchester UK

**Keywords:** Dialectical Behaviour Therapy, gay, Interpretative Phenomenological Analysis, lesbian

## Abstract

**Objectives:**

Lesbian and Gay people (LGP) experience higher rates of mental health difficulties, including self‐harm, suicidal behaviours, as well as inequalities in health care, than their heterosexual peers. Dialectical Behaviour Therapy (DBT) is an evidence‐based treatment for self‐harm and suicidal behaviours, though there is little research on LGP's experiences of DBT. This research aims to explore LGP's experiences of completing a full DBT programme.

**Design:**

A qualitative design with semi‐structured individual interviews was utilised. The results were analysed using Interpretative Phenomenological Analysis.

**Methods:**

Six lesbian and two gay adults, aged between 22 and 47 years, living in the United Kingdom took part. All participants had completed a full programme of DBT within the 2 years prior to the interview.

**Results:**

Four superordinate themes emerged from the data: (1) The DBT journey; (2) Connections and Sense of Community during DBT; (3) Sexuality both visible and invisible in DBT and (4) A Gender, Sexuality and Relationship Diverse (GSRD) affirmative future for DBT.

**Conclusions:**

Several clinical implications are suggested to improve DBT for LGP, for example to consider having other LGP within DBT groups, to create a more GSRD‐affirming DBT (changes to the DBT manual, DBT therapists, DBT programme and physical DBT space), to adapt DBT techniques to manage sexuality‐related difficulties and to adopt a GSRD‐centric framework. Overall, DBT appears to be beneficial for LGP.

## INTRODUCTION

Compared to their heterosexual peers, individuals identifying as Lesbian and Gay people (LGP) tend to exhibit elevated levels of psychological distress (King et al., 2008; Moagi et al., 2021), diminished mental well‐being (Semlyen et al., 2016; Woodhead et al., 2016), increased anxiety (Jones et al., 2017; Office of National Statistics, 2023; Stonewall, 2018), substance misuse (Woodhead et al., 2016); and discrimination (Moagi et al., 2021). LGP also experience higher rates of suicidal ideation, non‐suicidal self‐injury and risk of suicide than heterosexual individuals (Jackman et al., 2016; King et al., 2008; Swannell et al., 2016).

The challenges encountered by LGP in the United Kingdom are complex (Dunlop & Lea, 2023a, 2023b) and may be understood using Minority Stress Theory (Meyer, 1995, 2003, 2010), which is a key framework, advanced from Brooks' (1981) theory of sexual minority stress. Minority Stress Theory discusses how marginalised groups in society experience specific stressors related to their sexuality minority identity, predominantly due to prejudice and discrimination, which leads to increased mental health difficulties. Minority Stress Theory has been used to understand the unique stressors experienced by sexually diverse people (Meyer, 2003); gender diverse people (Hendricks & Testa, 2012; Meyer, 2015); and LGP of the Global Majority (Meyer, 2010).

Dialectical Behaviour Therapy (DBT) (Linehan, 1993, 2015) is an evidence‐based third wave cognitive behavioural therapy originally developed to treat people with a diagnosis of Borderline Personality Disorder,^1^ with a specific focus on self‐harm and suicidal behaviours (DeCou et al., 2019; Miga et al., 2019; Oud et al., 2018; Panos et al., 2014; Swales, 2020). Linehan (1993) developed the biosocial theory to explain the development of difficulties associated with Borderline Personality Disorder, suggesting that some people have high sensitivity and reactivity to emotional experiences with a slow return to emotional baseline, which, when coupled with an invalidating environment, creates emotional, relational and identity‐related problems to survive. Traditional DBT has four treatment modalities (see Table 1). Existing studies that have explored individuals' experiences of DBT have discussed participants' lives before DBT, relationships that supported change, developing self‐efficacy, a shift in perspectives, DBT causing positive changes, better control of emotions and improved relationships with others (Gillespie et al., 2022; Little et al., 2018).

**TABLE 1:** Standard DBT criteria.

There are four modes of treatment in DBT: individual therapy, group skills training, DBT consult team meetings and in‐between session contact between therapist and client (usually phone) Full course of standard adult DBT (Linehan, 1993, 2015), which fits the following criteria: Minimum 6 months, though ideally 1 year The structure of standard DBT involves a pre‐treatment period of time Therapist and client agree to work together A typical DBT agreement is 6 months or 1 year for adults Weekly individual and group sessions Diary cards, behavioural chain analysis, solution analysis and behavioural rehearsal OR Have completed standard adolescent DBT, if you were under the age of 18 during completion of your DBT course. Adolescent DBT is defined as (Rathus & Miller model, 2015): 16 weeks minimum, though usually 24 weeks The structure of standard DBT involves a pre‐treatment period of time Therapist and client agree to work together Weekly individual and group sessions Diary cards, behavioural chain analysis, solution analysis and behavioural rehearsal

Abbreviation: DBT, Dialectical Behaviour Therapy.

Given the significant minority stressors experienced by LGP, which lead to significant mental health difficulties, for example self‐harm and suicidal behaviour, DBT could be especially helpful for LGP. The literature discusses how the principles and skills within DBT apply to a Gender, Sexuality and Relationship Diverse (GSRD) population, for example emotional regulation seeks to support individuals in lessening susceptibility to distressing emotions whilst also adjusting and gaining control of them (Camp, 2023; Camp et al., 2023; Linehan, 2015; Pantalone et al., 2019; Skerven et al., 2019). Camp (2023) argues that, emotional regulation may help GSRD individuals lessen susceptibility to painful emotions that are a result of minority stressors (e.g. homophobia). The authors also argue that much like Minority Stress Theory, the biosocial theory of DBT offers an explanation for increased mental health difficulties, self‐harm and suicidal ideation in GSRD individuals due to the increased likelihood of experiencing an invalidating environment due to sexual minority status (Camp, 2023; Camp et al., 2023; Pantalone et al., 2019; Skerven et al., 2019). DBT has been adapted for sexual minority veterans (please see Cohen et al., 2021; Skerven et al., 2021) by incorporating minority stress theory (e.g. creating handouts on types of minority stressors) and adapting DBT skills. For example, Cohen et al. (2021) discuss adapting the DBT technique ‘Check the facts’ to consider further negative experiences that are more specific to GSRD individuals, for example discrimination, isolation and differential treatment. Although these results were promising, there is a paucity of research.

LGP also face structural heterosexism^2^ and inequalities experienced within health care. Findings suggest that LGP felt unequal in health care due to professionals' lack of knowledge, identification of sexual orientation, discomfort in interactions, heterosexist attitudes and perceived judgement (McNeill et al., 2023). An alternative to heterosexism would be affirmative psychotherapies, which are described as ‘therapy that is culturally relevant and responsive to Lesbian, Gay, Bisexual and Trans clients’ (O'Shaughnessy & Speir, 2018, p. 4). A recent development of the affirmative framework is the GSRD paradigm, which focuses on therapies and therapists (i) practicing a commitment to social justice, (ii) integrating core sexuality and gender theories, (iii) having knowledge of contemporary sexology, (iv) practicing cultural humility and competence, (v) holding knowledge of specific adverse effects of oppression and (vi) being trauma informed (Davies & Neves, 2023). Based on this paradigm, Dunlop and Lea (2023a) proposed the Intersectional, Social and Systems‐based framework to guide psychological assessment and formulation for GSRD individuals. This approach views minority stress within a historical context, framing difficulties as responses to systemic homophobia and heterosexism rather than individual maladaptation. Furthermore, Diamond and Alley (2022) describe ‘Safety signalling’, which describes actions, behaviours and symbols that indicate an environment is safe for a GSRD individual, further contributing to the GSRD paradigm and affirmative therapies.

Within the limited effectiveness‐based studies, DBT has shown improvement in adolescent LGP (Camp et al., 2024), GRSD adolescents (Poon et al., 2022) and GSRD adults (Chang et al., 2023; Oshin et al., 2024). To the author's knowledge, there is one study investigating GSRD adolescents' experiences of DBT (Camp et al., 2023). Camp et al.'s (2023) study found participants wanted to focus on minority stressors and independently suggested DBT skills to adapt for this; however, there was inadequate support to do this. The study also found connection to other GSRD individuals was helpful, and there was not enough focus on GRSD‐related difficulties and applying DBT skills to help with this. This is particularly important to understand, given the high rates of mental health difficulties, self‐harm, suicidal behaviours, health inequalities and poorer experiences of care in LGP. Furthermore, due to this high level of need, GRSD clients are highly represented within DBT programmes (Camp et al., 2024; Chang et al., 2023).

The current IPA research aimed to explore the experience of LGP completing a full DBT programme:

1. To explore the self‐harm and suicidal behaviours that brought LG adults into DBT.
2. To explore the experience of their sexuality within the DBT treatment.
3. To explore the experience of working on self‐harm and suicidal behaviours within a full DBT programme to completion as a LG person.
4. To explore how participants experienced DBT as affirmative or not of their sexuality.

## METHOD

### Design

This research was a qualitative study, which utilised individual semi‐structured interviews to gather data. The research employed Interpretative Phenomenological Analysis (IPA; Smith & Osborn, 2003). IPA aims to thoroughly explore an individual's understanding of their own personal and social worlds with a focus on the significance that these events hold for an individual (Smith & Osborn, 2007). IPA enables the incorporation of extensive personal details and real‐life experiences (Larkin et al., 2006). Qualitative research, such as IPA, gives voice to marginalised groups to express their experiences when other academic methods give them less power (Booth, 2018).

### Ethics statement

A Full University Research Ethics Committee (UREC; ID:2022‐15235‐26394) review was undertaken by The University of Manchester and full ethical approval was granted.

### Participants

Purposive sampling was used to recruit participants who met the following inclusion criteria: (a) self‐identify sexuality as lesbian or gay; (b) aged between 18 and 50 years; (c) able to speak English fluently; (d) have a UK‐based GP; (e) consent to participating in a recorded interview; and (f) have completed a full course of standard DBT within the last 2 years (see Table 1).

The research team aimed to interview 8–10 participants, in line with the recommended sample size for IPA studies (Smith et al., 2009; Turpin et al., 1997). After eight interviews, the research team reached consensus that the data retrieved provided ample richness and detail sufficient to complete IPA (e.g. Hale et al., 2007). Furthermore, this maintained a small sample size to enable analysis of similarities and differences across the sample (Brocki & Wearden, 2006).

### Procedure

The study primarily used social media to recruit participants, which is an effective way to recruit participants from marginalised groups (Kayrouz et al., 2016). The study recruitment poster was distributed via X, Facebook and to relevant organisations, for example the National Survivor User Network (NSUN) and the LGBT Foundation. Prospective participants contacted the research team, and a screening call was undertaken to assess eligibility. Interviews were then conducted via Zoom (Version 5.17.1131580), lasting between 73 and 121 min.

Prior to the interview the following were obtained: audio‐recorded informed consent was obtained, demographics and participant personal information. A topic guide was collaboratively created by the research team and experts by experience. Participants were then offered a debrief, a well‐being check‐in call and signposting to relevant organisations. Participants received a £25 voucher.

### Analysis

The primary researcher (C.H.) transcribed interviews verbatim, anonymised participants and assigned pseudonym names. Smith and Osborn's (2007) four‐step process for IPA was utilised, line‐by‐line coding including linguistic and emotional observations. Emergent themes were identified and explored for connections and clustered into subordinate and superordinate ideas. Emergent super‐ordinate themes were repeatedly reconfigured and refined and compared against the raw data for accuracy. Smith and Osborn's (2007) approach of using the themes from the first transcript to directly analyse the other transcripts was utilised to help highlight similarities and differences between transcripts. Certain themes were prioritised based on prevalence, richness and importance relative to other themes.

### Trustworthiness and rigour

Guba and Lincoln's (1989) criteria to establish trustworthiness and rigour in qualitative research were followed: credibility, transferability, dependability and confirmability. To permit opportunities to examine and reflect on each analytic stage, researcher triangulation was utilised during the interview and developing analysis stages. To enrich the trustworthiness of the data, the second author (J.L.), an experienced IPA researcher, double‐coded a sample of transcripts. The research team agreed on the themes within this manuscript. Four participants engaged in ‘member‐checking’ by offering feedback on their personal interview analysis, and this supported the development of the themes. This process increases the analytic validity, trustworthiness and credibility of the findings (Birt et al., 2016; Goldblatt et al., 2011). An audit trail of analysis and analytical records to reflect on the research processes was also created.

### Reflexive positioning

The first author (C.H.) engaged in reflective practice to support reflexivity (Clancy, 2013), which is a definitive standard for determining trustworthiness (Teh & Lek, 2018). C.H. is a heterosexual British Filipina and trainee clinical psychologist, with no formal DBT training. J.L. is a White British, Queer, consultant clinical psychologist, academic, DBT therapist and accredited DBT supervisor. D.P. is a White British, heterosexual, consultant clinical psychologist and academic.

The research team continuously reflected on their epistemological positions. Some argue that heterosexual researchers cannot contribute equally to GSRD research compared to GSRD peers (Schlichter, 2004; Thomas, 2000). Others argue identity does not dictate research outcomes and that heteronormative knowledge stems from entrenched power dynamics (Allen, 2010). It is important to note that all three authors were concerned with improving DBT for LGP, therefore remaining aware of their personal positions throughout.

## RESULTS

### Participants characteristics

Fifteen people contacted the study email address to take part. However, some did not meet inclusion criteria (*n* = 5) and some disengaged (*n* = 2). Eight people participated in the study, aged between 22 and 47 years (*M =* 31.13). Some participants had intersectional identities, for example a participant who identified as a woman, unemployed and a lesbian. Participants either attended a GSRD affirming programme, a majority GSRD individuals programme or a DBT programme that was not GSRD majority. See Table 2 for more details.

**TABLE 2:** Participant characteristics.

Pseudonym	Gender	Sexuality	Age	Ethnicity	Religion	Education	Employment and study	DBT	GSRD affirming/majority/non‐majority Programme
Tom	Male	Gay	26	White British	Christian	Undergraduate	Part time work and studying	Adult	Non‐majority
Evelyn	Female	Lesbian	41	Mixed Ethnic Origin	None	Postgraduate	Unemployed	Adult	GSRD majority
Felicity	Female	Lesbian	28	White British	None	Postgraduate	Employed	Adult	GSRD affirming
Georgia	Female	Lesbian	23	White British	None	Postgraduate certificate in education	Employed	Adult	GSRD majority
Jacob	Male	Gay	27	Mixed Black Caribbean and White	None	Postgraduate	Part time work and studying	Adult	Non‐majority
Racheal	Female	Lesbian	22	White British	None	GCSE'S	Part time work and studying	Adult	GSRD majority
Lydia	Female	Lesbian	47	White British	Christian	Undergraduate	Unemployed	Adult	GSRD majority
Yolanda	Female	Lesbian	35	Preferred not to answer	Preferred not to answer	Preferred not to answer	Preferred not to answer	Adult	Non‐majority

Abbreviations: DBT, Dialectical Behaviour Therapy; GSRD, Gender, Sexuality and Relationship Diverse.

### Interpretative phenomenological analysis

**FIGURE 1:**
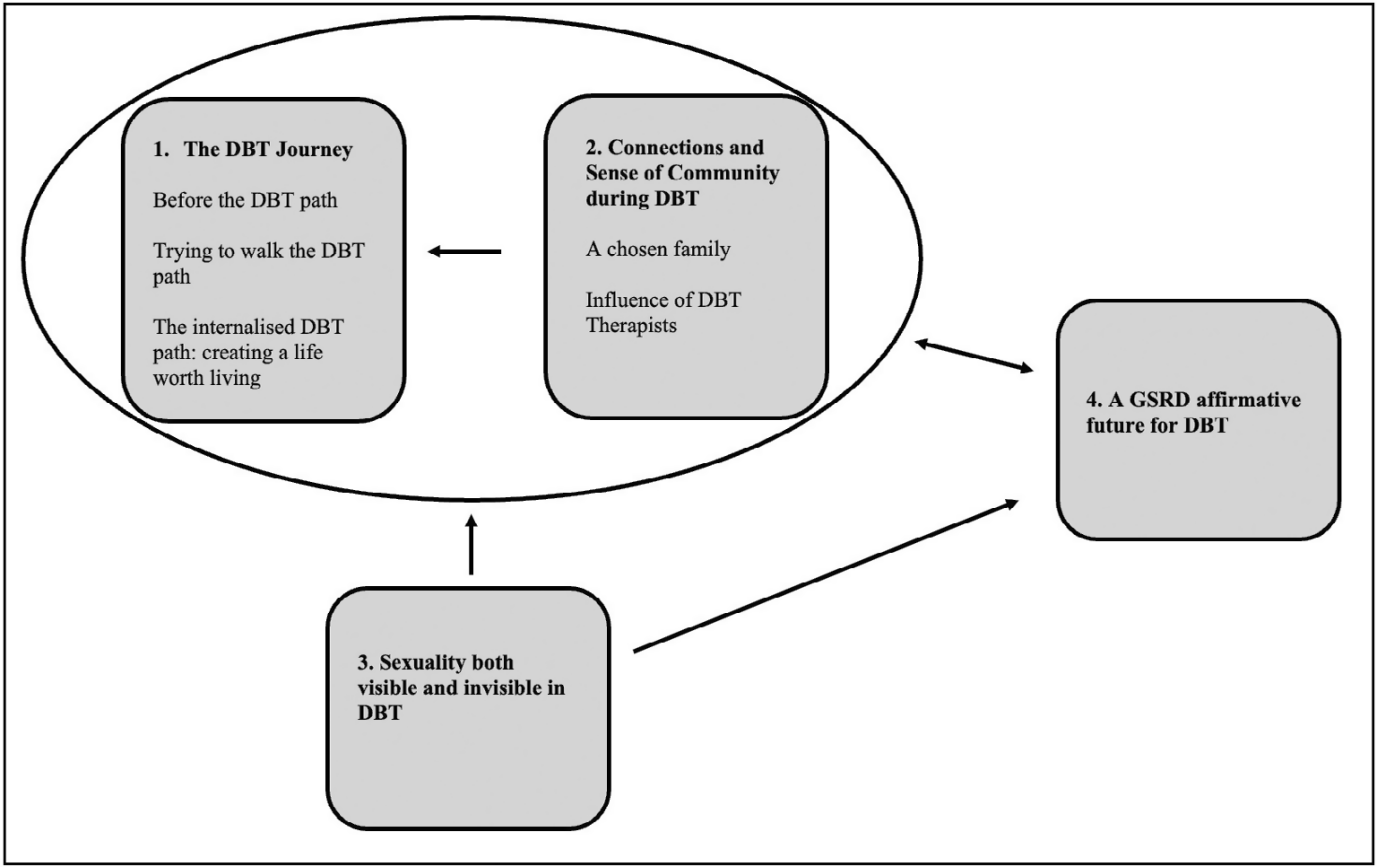
The relationships between superordinate and subordinate themes.

Four superordinate themes and five subordinate themes emerged from the analysis (see Figure 1 for a diagrammatic representation). All participant names are pseudonyms.

### The DBT journey

All participants endeavoured on a journey before, during and at the end of their DBT programme. All participants had a difficult journey to finding DBT, but participant experiences whilst engaging with DBT were varied, and not all participants ended with creating a life worth living.

#### Before the DBT path

Participants described a difficult path to finding DBT whilst trying to live with longstanding difficulties, such as Tom who ‘was struggling a lot with things like anxiety, depression, self‐harm’. Participants spoke of self‐harm and suicidal ideation with a particular pain and frustration.

I went to my GP and said this is it I have reached crisis point … either you detain me or um or I will kill myself because I just can't fight the compulsion to kill myself anymore. (Evelyn)

The desperation for a solution and reaching out for help was palpable in participants words.

When I self‐harmed and went to A&E or other services they were really bad and that just triggered me to do it more. (Yolanda)

This suggests participants felt unheard by services, potentially exacerbating health care inequalities for LGP. Participants' ongoing struggles with suicidal ideation and/or self‐harm were often linked to their sexuality‐related difficulties, further contributing to suicidal ideation:

I was bullied quite a lot at school because of my sexuality. (Jacob)

Like around identity and sexuality and feeling like I didn't belong anywhere sort of, it created a lot of suicidal thoughts. (Tom)

Participants had often experienced various therapies prior to DBT, which appeared inadequate for their complex needs. Participants spoke about past therapies with an undertone of frustration and annoyance:

I find that with CBT sometimes what you end up doing is putting a plaster over it and just leaving it and then it reopens again. (Georgia)

I found a charity that offered low‐cost counselling or whatever it's called, so I went for that and that actually ended up being worse. (Jacob)

It often felt that when DBT was offered, participants felt like they had exhausted every avenue of therapy, and saw it as another therapy that would be unsuccessful:

I'm gonna go to these appointments to prove a point, to show them that it doesn't work. (Racheal)

Participants seemed to have lost hope in therapies and appeared to engage with DBT to fulfil others' wants rather than their own. This may reflect the inequalities LGP face within health care, which may lead to unfavourable perspectives of therapies. Overall, all participants had experienced arduous journeys, which led them to DBT.

#### Trying to walk the DBT path

Much like the DBT skill ‘walking the middle path’, participants seemed to experience different dialectical extremes throughout DBT, making it challenging to remain on the DBT path. Initially, it appeared participants struggled to socialise themselves with the model, finding the strict structure difficult.

It just felt like being back at school and the rules, if you didn't do something or bring something to session you had to talk about what got in the way. (Jacob)

Due to past therapy experiences, participants struggled with the focus on skills and felt frustrated that ‘you couldn't just talk’ (Lydia). Other participants struggled with the specifics of the DBT manual, for example it was experienced as ‘fiddly’ (Jacob), ‘patronising’ (Felicity), or ‘so American’ (Lydia), further increasing difficulties with socialisation to DBT.

Most participants also struggled with the specific DBT techniques used. For example, many discussed difficulties with completing diary cards^3^ as this was seen as ‘a redundant exercise’ (Evelyn), suggesting that participants may not have grasped the purpose of diary cards. Some spoke about the experience as quite overwhelming:

I didn't do it every day cause it was just a lot. (Jacob)

Seeing that written down was quite hard. (Georgia)

For some it was more the practicalities: ‘There was never much room to write about what actually happened on that day’ (Lydia). Due to all these factors, some participants seemed to avoid the technique, demonstrating a tendency to disengage when a technique was difficult. The avoidance of techniques may also be explained by participants scepticism around the DBT model. For example, some participants remained cynical about techniques such as ‘The Wise Mind’^4^ appearing to not ‘buy‐in’ to the DBT model, causing confusion:

Just because I'm responding to something emotionally doesn't mean that that emotion in itself is irrational or unreasonable. (Felicity)

That just baffled me I was like I can't access another part of my mind as I'm just living in emotion mind. (Lydia)

Some participants also discussed struggles with engaging with the programme whilst experiencing heightened emotions and suicidal ideation and/or self‐harm urges, which sometimes contributed to avoidance:

I guess sometimes I wasn't in a good head space and wasn't able to engage. (Tom)

Conversely, DBT seemed to serve as a welcome blockade to suicidal ideation and/or self‐harm to others:

It was a healthy form of distraction. (Evelyn)

It was something that would come into my mind, and I want to do that but now I can't do that. (Georgia)

After the initial struggle to socialise with DBT, participants began to ‘walk the middle path’ and started to see benefits to techniques. For example, Evelyn, Felicity, Georgia and Lydia all found benefit from using the diary cards once they started committing to them:

When I did them properly, it sounds silly now, but they were actually quite useful. (Lydia)

Many participants discussed the helpfulness of specific techniques and modules. For example, many participants resonated with DBT's biosocial theory of emotional regulation, which appeared to be experienced as normalising:

It allowed me to become more aware of my thoughts and feelings and reasons to why I might get triggered by things and why I get stressed at certain situations (Tom)

It makes a lot of sense in terms of my upbringing and why I am the way I am now. (Jacob)

Alternatively, participants noted things that they did not find helpful. However, participants appeared to reflect on this and conclude that although not everything resonated with them or felt beneficial, they were able to take a lot from the programme therefore walking the middle path:

It's a bit like trial and error I guess, um you know there's some things which, um some tools which I didn't find useful and that aren't as applicable for people. (Tom)

It's a lot of commitment and it's a long time and it kind of teaches you that progress is not something that is linear, it takes time and there will be ups and downs. (Georgia)

#### The internalised DBT path: Creating a life worth living

Most participants experienced an empowering end to DBT, concluding with creating a life worth living. Many participants praised DBT for the changes that it had helped them facilitate in their lives, appearing to transition from their original scepticism. Many participants discussed an increased awareness of their emotions and behaviours, which led to a greater sense of autonomy, appearing to be a lifeline for some:

In a way gaining distance and a healthy sense of detachment from my emotions … you know it's that line from, from uh dune, after fear has passed only, I will remain, so it's like all the horrible emotions have passed I still remained, and I was safe. (Evelyn)

It's so empowering actually be able to understand at least to some degree the sort of external and internal factors that might lead you to behave or react a certain way. There's a certain degree of control over that. That's something that was genuinely life changing for me. (Felicity)

Some participants discussed feelings of increased confidence in their identities, which was something most struggled with pre‐DBT:

It allowed me to go this is who I am. But I think that was part of me going I am who I am and I need to start liking and respecting myself and being confident in who I am in terms of I'm a lesbian and I'm a single mum. (Lydia)

Although participants noted a reduction in suicidal ideation and/or self‐harm urges, it appeared there was a level of disappointment that they had not completely faded:

It definitely helped me keep away from self‐harm and suicidal thoughts. (Tom)

I still have urges now … that towards the end of the therapy I felt a bit like … my expectation was that they are supposed to go away. (Jacob)

It appeared that many participants anticipated suicidal ideation and/or self‐harm urges to completely dissipate on completing DBT, suggesting they had invested hope within DBT to facilitate this change. However, it seemed that many participants ended their DBT journey with a sense of accomplishment:

I didn't think life was worth changing and now at the other end I'm like, difference is obvious. (Lydia)

Participants were able to transition from experiencing severe mental health difficulties entangled with difficulties related to sexuality and self‐harm and/or suicidal ideation to a significant reduction in these difficulties. It appeared participants had found renewed hope for the future, marking the start of a new chapter in their lives. However, for some, there appeared to be barriers within trying to walk the DBT path that reduced their ability to find a life worth living:

I didn't get any relief, it's quite the opposite, when someone talks to me about that and that wow, I just felt so overwhelmed. (Yolanda)

### Connections and sense of community during DBT

Some participants discussed a felt sense of community that formed throughout their DBT programme, whereas others discussed a lack of this feeling. The level of community experienced impacted on how participants felt about the DBT programme. Furthermore, participants discussed their connection (or lack of) to DBT therapists.

#### A chosen family

Participants within GSRD majority programmes formed a chosen family with other DBT participants:

We kind of found a bit of a kinship with each other because we were able to relate to the fact that we'd all had the same experience and that had contributed to how we were feeling now. (Georgia)

Participants often found ‘A chosen family’ most strongly with other LGP in their DBT groups. Being in the DBT groups seemed to be experienced as normalising for participant's difficulties:

In the broader sense being able to draw on their own experiences and say yep, I have experienced something like that, and this is how I dealt with it. Or this is a really familiar feeling to me, being able to emphasise in that way. I think that was really really helpful. (Felicity)

Unfortunately, for some participants, there was a lack of sense of community and family throughout their DBT experience. For some, the group set up felt overwhelming, and it appeared difficult to share personal experiences with people who were considered strangers.

Sometimes other people's stuff would trigger me as it related to me and that wasn't easy. (Yolanda)

There are certain expectations that you bring examples from your life, but a lot of my experiences are very coded of being gay … I didn't feel comfortable talking about my sexuality in the group. (Jacob)

It felt as though Yolanda and Jacob were unable to bring their true self to their DBT programmes; in turn, they were unable to form a chosen family with other DBT members. Interestingly, Jacob and Yolanda also struggled to internalise the DBT path. This may show that people's experience of the DBT group may impact their overall perception of DBT. The majority found value in their chosen DBT families, and some continued to seek support from chosen families post‐DBT, demonstrating the strength of these connections:

I'm still in touch with some of the people I was in therapy with so that's quite a good resource … we support each other. (Lydia)

#### Influence of DBT therapists

Nearly all participants discussed the influence of the DBT therapists. A vital theme impacting on connection to therapists was listening skills:

This guy, this therapist sat through and listened to every fucked … every fucked up thought I ever had and said that makes total sense and it makes sense that you feel that way and here is why you might. (Felicity)

He was always saying like whatever you say, there's no judgement here. (Racheal)

When therapists actively listened, participants appeared to feel validated and able to discuss more personal topics, which in turn felt more valuable. However, in some instances, therapists did not display this. For example, when Racheal was discussing a sexual microaggression:

She turned round, and she said, I'm confused now, do you … were you hit by a man or a woman, and I was like, were you not listening. (Racheal)

It felt as though this was quite invalidating for participants, especially when disclosing difficult experiences. Participants also discussed how therapist disclosure positively impacted their connection to the therapist. Furthermore, if a therapist disclosed their shared minority sexuality, this appeared to build a therapeutic alliance and may have increased a sense of safety to discuss sexuality‐related topics.

He kinda shared a lot more about himself, so I was like able to connect more personally on a more personal level. (Tom)

One of them shared that she was LGBTQ+ as well which kind of helped a little bit because it was like oh okay, we have some similarities here. (Georgia)

For others, therapist disclosure seemed to be invalidating and depreciated the participants own experience's:

Not trauma dumping but she just talked about all the stuff her brother had gone through which sounds traumatic. So, I was sat there like hm yeah cool like. (Jacob)

For many participants, the availability of their therapist was important to them. Lydia compared her DBT therapists: ‘Being able to message Anna and say this has happened can somebody contact me, for me was really important and it really annoyed me about Janice my previous one to one … I had no way of contacting her outside of our sessions, other than through the group, The DBT inbox’ (Lydia). It appeared that therapist availability had an impact on the ability to form a therapeutic connection, as Lydia continued:

I liked Janice, and I did build a rapport with her, but it wasn't as easy as it is with Anna. (Lydia)

However, connection to therapists also seemed to depend on the therapists' personal attributes, for example their use of ‘*humour*’ (Jacob, Evelyn) and being ‘*easy going and chilled*’ (Lydia). It appeared that the more personable DBT therapists presented as, this increased the sense of connection and influence for participants:

There was no silver bullet, there was nothing specific he said, he didn't turn around and come out with this magical line that made me want to live again. I can't remember much of what he said at all, but he turned up and um, you know he cared about me. (Felicity)

### Sexuality both visible and invisible in DBT

Excluding the GSRD affirming DBT programme there was a general sense that discussing sexuality was not a concern, despite participants describing sexuality‐related difficulties as a contributor to mental health difficulties, self‐harm and/or suicidal ideation. Participants often felt the topic was overlooked and was not considered within DBT:

I kinda feel like it was like little bits that were brushed under the carpet, yeah I'm not really sure if like the therapists in the team felt comfortable talking about it (Tom)

It appeared that this reduced participants' confidence in their DBT team to discuss sexuality‐related difficulties. Participants found themselves receiving generalised/problematic statements surrounding sexuality, which could often be deemed subtly oppressive and negate participants' feelings:

She kept saying “oh my beliefs are that everyone should be treated equally no matter if they're blue, yellow, gay” … it just felt like a blanket response that was going over what I was actually talking about. (Jacob)

There were often times where participants continuously asked for support, regarding their sexualities, but this question appeared unmet:

All of a sudden someone would go oh what about homosexual people and it was just like we had to ask for it to be brought up and when it was very superficial. (Georgia)

It appears that this was not addressed within DBT and would lead participants to question why. In some cases, DBT appeared to be experienced as heteronormative. For example, participants described suggestions around stereotypically cisgendered activities, which seems to uphold the gender biases, which appeared quite frustrating for one participant:

They would say things about kind of for the girls like going out with your friends or doing this or doing that and with the boys it was like go and play football. I remember thinking like, read the room. (Georgia)

One participant also stated: ‘There were no affirmative text about being gay’ (Jacob). This suggested that DBT still privileged heterosexuality, as no adjustments were made to include GSRD examples, reinforcing heteronormativity within the health care system. Some participants felt DBT overlooked sexuality within theories and techniques, therefore missing opportunities to address the unique difficulties faced by LGP, which may have hindered participants opportunities to understand sexuality‐related difficulties. Felicity made several comments about radical acceptance,^5^ the biosocial model and dialectical thinking:

I still do struggle with that the idea that in the radical acceptance skill where accepting something radically all the way completely, you're not necessarily condoning it … as a lesbian woman I think I know that unless you're actively working to change a system of homophobia or actively working to change a system that excludes people you are condoning the status quo. (Felicity)

However, Georgia and Jacob felt they were able to apply some specific DBT techniques to the minority stressors they faced due to their sexuality. For Jacob:

The paced breathing has been quite helpful sort of before leaving the house sometimes I use it, if I'm going out with friends, a gay event, obviously I want to dress how I want to dress but sometimes I can't leave the house. (Jacob)

Jacob was able to apply a DBT skill to help calm his fear about being discriminated against out in public, and he was able to adapt DBT for this. In Georgia's case, her DBT group therapist was the one who made suggestions around managing difficulties with sexual identity:

One of the main things that they kept saying to us was to seek validation from ourselves and seek it from within. (Georgia)

However, only Georgia stated her DBT therapists provided her with techniques to manage sexuality‐related difficulties, yet she still expressed frustration that not enough was done to consider her sexuality throughout DBT. Although sexuality was overlooked within DBT, some participants were accepting of this due to prioritising other difficulties:

It wasn't kind of uh very extensive around sexuality because …. It was more around how to keep myself alive really and to keep myself safe. (Yolanda)

### A GSRD affirmative future for DBT

All participants discussed recommendations that would make DBT a better experience for them as LGP, which suggested working towards an GSRD affirming DBT programme. Many participants referred to having an ‘open’ (Evelyn) and ‘non‐judgemental’ (Tom) environment and the importance of reiterating this:

As far as sexuality I think I supressed that a little bit in myself and maybe the therapist could be clearer that it is a very open platform, and you don't really need to hide anything. (Lydia)

It felt as though, if therapists were more reassuring around sexuality disclosures, participants may have felt more about to share sexuality‐related difficulties, therefore creating a more validating experience. For Yolanda, this would mean a focus on confidentiality:

Confidentiality is a big thing and it's about creating a safe space, so people are able to talk. (Yolanda)

Georgia and Jacob stated gestures that make their environment feel more comfortable and a safer space, for example ‘pride progress pins’ (Georgia, Jacob), ‘pride flags’ (Georgia), ‘lanyards with rainbows on’ (Jacob). They both discussed how these actions made them feel more comfortable within DBT:

Gives the vibe that I can talk comfortably about sexuality. (Jacob)

At times, it appeared as though DBT therapists attempted to be more GSRD affirming, although their efforts were not consistent. This then seemed to be experienced as unfamiliar and awkward:

We always would go round and introduce ourselves, never before have facilitators said could you tell us your pronouns … I remember that feeling really uncomfortable that day. (Lydia)

Interestingly, participants who attended GSRD majority DBT programmes, which by virtue of participants identities were more open and accepting, spoke of the benefits of having a group with majority GSRD people:

I'm saying I got lucky, but I think actually having a queer affirming or an LGBT affirming group was crucial to how I engaged with the program. (Felicity)

I found it safer to do it in a group with other people within the LGBTQ+ community. (Evelyn)

It presented as normalising and validating to share experiences with other GSRD people. Those who did not attend majority GSRD DBT programmes made recommendations around DBT programmes being grouped GSRD majority:

Having a DBT for people who identify specifically as LGBT+, I think that would be 10 times better for me, because I feel like I could at least come to the group as I am. (Jacob)

It seemed that having this experience may have given participants more confidence to share difficulties in relation to their sexualities and form connections to others. This is specifically important for Yolanda and Jacob, who did not find a chosen family within DBT. Many believed sexuality‐related difficulties were unnoticed and suggested taking time to discuss this as beneficial:

I'm not saying have a whole module on being a lesbian or whatever, but I think talking about some of those factors. (Lydia)

Making a session like specifically about like how you identify and like any troubles that you've had with it. (Racheal)

It appeared this would have made participants feel as though their sexualities were acknowledged and sexuality‐related difficulties were heard. Tom and Yolanda discussed having lived experience with therapists or therapists to have specialist training around sexuality:

Have training around like how to talk more openly and more comfortably around topics around sexuality and identification. (Tom)

It seemed that having more experienced therapists would increase participants' confidence in their therapists to discuss sexuality‐related difficulties. Having all these things in place, participants believed would create a more open and affirming therapy space, which would all suggest a GSRD affirming DBT.

## DISCUSSION

This study sought to understand the experiences of eight LGP who had completed a DBT programme using IPA. The primary themes identified were The DBT journey, Connections and Sense of Community during DBT, Sexuality both visible and invisible in DBT, and A GSRD affirmative future for DBT and five subordinate themes were: before the DBT path, trying to walk the DBT path, The internalised DBT path: creating a life worth living, a chosen family and Influence of DBT Therapists. To the authors knowledge, this is the first IPA study that specifically explores LGP's experiences of completing a full DBT programme.

LGP's experiences pre‐DBT mirrored findings of studies on DBT experiences that did not focus on sexuality, for example finding a lack of hope for the future and a sense of distress (Hodgetts et al., 2007; Little et al., 2018). Similarly, studies found that individuals did not feel understood or unsupported by services (Hodgetts et al., 2007; Little et al., 2018). LGP also discussed inequalities experienced in health care, in line with past research (McNeill et al., 2023).

Past research also found similarities around individual's experiences of trying to walk the DBT path. For example, adolescents found DBT language hard to understand, but then became familiarised with it (Lakeman & Emeleus, 2020). Barnicot et al. (2015) found individuals struggled to understand the materials and found the skills hard to practice but then became socialised to the model. In line with past research many participants were understanding of the difficulties of DBT and the need to engage to make progress (Gillespie et al., 2022).

Congruent with past research, many participants were able to create a life worth living and found DBT life changing (Cunningham et al., 2004; Ohlis et al., 2023). Participants experienced increased control, strengthened identities and hope for the future, parallel to past research (Cunningham et al., 2004; Gillespie et al., 2022; Little et al., 2018).

The finding around strengthened identities is especially important, as lower levels of acceptance around sexuality (e.g. self‐acceptance, family/friend acceptance and internalised homophobia) are associated with higher levels of minority stressors, mental health difficulties and lower general well‐being for GSRD individuals (Camp et al., 2020). Therefore, self‐acceptance could be an important target for LGP in DBT. Nevertheless, a minority did not appear to internalise the DBT path. It appeared this may have been due to a lack of connection to the group and/or therapist. Furthermore, research has suggested participants ‘conquered’ their self‐harm and/or suicidal ideation, whereas the current study found some participants still struggled with self‐harm and/or suicidal ideation. This may result from the additional difficulties LGP face compared to heterosexual peers, leading to increased levels of self‐harm and suicidal ideation (King et al., 2008).

LGP often face discrimination and prejudice from their family systems, resulting in frayed relationships and diminished support. Often this leads to forming a chosen or logical (rather than biological) family. Some participants found connection to others due to their shared sexual minority identities, which increased engagement with DBT, consistent with past research (Barnicot et al., 2015; Ceatha et al., 2019). Furthermore, this finding aligns with Camp et al.'s (2023) study exploring GSRD adolescents' experiences of DBT, where participants found connection to each other, which was experienced as normalising and increased support. Meyer's Minority Stress Theory (Meyer, 1995, 2003, 2010) states that coping and social support can act as moderators for sexual minorities mental health. Meyer (2015) later goes on to state that resilience is also an important protective factor that comes hand in hand with coping, which also mitigates the impact of minority stressors. Therefore, this finding is important as social connectedness helps build resilience, which has a positive impact on well‐being for LGP (Ceatha et al., 2019; Detrie & Lease, 2007; Garcia et al., 2020; Joyce et al., 2024), especially as social connectedness is something that LGP can struggle with (Meyer, 2003). Participants who did not attend a GSRD affirming/majority programme struggled to find social support through their DBT groups, in turn leading to a more negative experience of DBT. This highlights the importance of having other LGP within DBT to form the social support needed to engage with the programme but also to decrease psychological distress (Joyce et al., 2024) and protect against the negative impacts of minority stressors.

The DBT therapists influence often shaped how participants experienced DBT. ‘Higher quality’ therapist disclosure, for example around shared sexual minority status, insight and feelings, was considered valuable and increased the therapeutic alliance, corresponding to past research (Lea et al., 2010; Pinto‐Coelho et al., 2016; Porter et al., 2015). However, sexuality‐related therapist disclosure can be perceived as unhelpful when it is for therapist benefit (Lea et al., 2010). Furthermore, this links to the GSRD paradigm (Davies & Neves, 2023), which states that therapists can share lived experiences if this is perceived as helpful, for example shared social support or local knowledge. However, the GSRD paradigm also states that it is not essential, and the client should act as a co‐therapist and teach the therapist about their beliefs and communities (Davies & Neves, 2023). The therapist's humour was an important factor in creating connections, which is fundamental within DBT (Buerger & Miller, 2022), and helped increase therapeutic alliance. This may have led to therapists being perceived as more approachable, therefore creating a protective space for behaviours to be challenged (Brooks et al., 2021). Overall leading to increased therapeutic alliance, which was demonstrated within the current study. It is important to note that there were times where participants felt unheard. However, many participants discussed therapists' skills to listen, which made them feel validated, cared for and heard, contradicting previous research on health care inequalities (McNeill et al., 2023). This suggests participants perceived DBT more positively than other health care providers.

Within the theme ‘Sexuality both visible and invisible in DBT’, the DBT manual was experienced as heteronormative due to cisgendered heterosexual examples and no sexuality affirming text, leading to sexuality feeling invisible at times. Findings suggest that DBT therapists may lack confidence in addressing sexuality‐related difficulties, leading to these aspects being overlooked in therapy. In the current study, it appeared that most clients' needs around sexuality were unmet, which mirrors past research demonstrating inequalities in health care for LGP (McNeill et al., 2023). Additionally, clients reported therapists' using heteronormative statements, considered microaggressions, resulting in weakened therapeutic alliances and decreased therapy effectiveness (Spengler et al., 2016). However, one participant attended a GSRD affirming DBT programme, which demonstrates an effort to increase visibility of sexuality. For example, within the GSRD affirming DBT programme, everyone identified as GSRD. Moreover, some participants were able to successfully apply DBT techniques to sexuality‐related difficulties. The research base around DBT for LGP is in its infancy, and practitioners were unlikely to be aware of adaptions that could be made. Although difficulties around sexuality were mostly unmet, most participants were able to improve their mental health and some found their sexual identities strengthened. This may link back to Minority Stress Theory (Meyer, 1995, 2003, 2010), which stipulates that social support can act as a mediator to mental health difficulties, which is demonstrated for some within the current study. This theme was similar to Camp et al. (2023) study, where participants reported frustrations around a lack of GSRD‐related topics and having inadequate support to apply DBT techniques to GSRD‐related difficulties. However, similar to the current study, participants saw the potential in the DBT skills, and some were able to apply these to GSRD‐related stressors.

Participants described a GSRD‐affirming future for DBT, in line with the GSRD paradigm (Davies & Neves, 2023). Recommendations from participants mirror research regarding GSRD individuals and DBT, for example GSRD‐specific modules/techniques, specialist training for staff and GSRD examples (Camp, 2023; Camp et al., 2023; Pantalone et al., 2019; Skerven et al., 2019), further strengthening the argument for GSRD affirming DBT. Furthermore, participants recommended an open and non‐judgemental environment, further in line with Camp and colleagues' study (Camp et al., 2023). Participants also discussed concerns around confidentiality and the need for safety signalling (use of pride flags and pins, etc.), in line with Diamond and Alley (2022). This finding further demonstrates the inequalities in health care experienced by LGP, as for most participants, sexuality was overlooked.

### Limitations and future research

Six participants were lesbian women; therefore, the views of men were not as represented. More selective sampling may have supported valuable insights into the experiences of gay men completing a DBT programme. Additionally, the views of young people (18–21 years) were not represented, nor were the views of people aged 47 and over. Furthermore, due to the sampling strategy, the study is likely to include more views of enthusiastic participants who wish to discuss their experiences, those who have access to the Internet and those who speak English.

As difficulties for other GSRD individuals are like LGP's (Dunlop & Lea, 2023a), there should be further research into these groups' experiences of DBT. Furthermore, research focusing on experiences of GSRD individuals completing a DBT programme advertised as GSRD affirmative would be an important avenue for research. This may provide further clinical implications, which can be implemented.

### Clinical implications

Many participants found having other LGP within their DBT group valuable. Firstly, it is recommended that LGP can participate in DBT groups with other LGP, aligning with past research (Camp et al., 2023). This may be through attending a GSRD affirmative DBT or a general DBT programme (e.g. Cohen et al., 2021; Skerven et al., 2019). Secondly, participants discussed working towards a more GSRD affirmative DBT. This included recommendations around the therapy environment, the DBT manual, the DBT programme and therapists. Table 3 includes recommendations around creating a more GSRD affirmative DBT, informed by the current study and past research (British Psychological Society, 2019; Camp, 2023; Camp et al., 2023; Davies & Neves, 2023; Freeman‐Coppadge & Langroudi, 2021; Hudson & Bruce‐Miller, 2023; McClain et al., 2016; Pantalone et al., 2019). One notable recommendation within the table is GSRD therapists, as the current study found a closer alliance was built when there was a shared sexual minority status. However, it is crucial for DBT therapists to consider when sexuality minority status disclosure will or will not strengthen the relationship.

**TABLE 3:** Clinical recommendations around GSRD Affirmative DBT.

Adaption	Action
Creating a more physically affirming GSRD space	Create a welcoming environment for GSRD people e.g. pride pins, pride lanyards and GSRD‐affirming posters etc. Use of pronouns (displayed next to names if online)
DBT manual	Examples of same‐sex relationships and families LG affirmative text Link biosocial theory and Minority Stress Theory Case studies that related to LG mental health difficulties Use inclusive GSRD language through the manual e.g. instead of saying ‘men and women’, say ‘all genders’
DBT programme	Reviewing group guidelines to include discussions around gender, sexuality and respect Being in a DBT group with other LGP A module/section to discuss difficulties around identifying as a sexual minority and techniques that may help with this Creating a confidential space for individuals e.g. reiterating confidentiality in terms of someone's sexuality An open space to get things wrong and learn e.g. being curious to learn about someone's sexuality Icebreakers around sexuality
DBT therapists	Additional training on the difficulties faced by sexual minorities Remain non‐judgmental and compassionate to sexuality‐related difficulties GSRD DBT Therapists Therapist self‐disclosure of sexuality (used with caution) Therapist's stress that it is an open platform where clients can share, including sexuality. Use of humour Use of inclusive language specific to minoritised sexuality experience.

Abbreviations: DBT, Dialectical Behaviour Therapy; GSRD, Gender, Sexuality and Relationship Diverse; LGP, Lesbian and Gay people.

Additionally, clinicians should consider techniques to help LGP with minority stressors. DBT's biosocial model explaining the difficulties experienced by people that can lead to self‐harm and suicidal behaviours could more clearly highlight the similarities between an invalidating environment (Linehan, 1993) and Minority Stress Theory (Meyer, 2003) to develop a theory specific to GSRD people receiving treatment in a DBT programme (Camp, 2023; Skerven et al., 2019). This formulation‐based approach could support GSRD people understand and relate to their difficulties without being pathologised and medicalised (Dunlop & Lea, 2023b). It has also been suggested that in creating a life worth living, one may need to radically accept that we did not create homophobia or heterosexism but still must address it for our personal and collective well‐being.

Though the research base is still young, there are some important recommendations around adapting DBT for LGP (Camp, 2023; Camp et al., 2023; Pantalone et al., 2019; Skerven et al., 2019). For example, in the current study, one participant described an experience of a sexual microaggression, so it could be suggested she focuses on self‐sooth, recovering from invalidation, objective effectiveness and checking the facts (Camp et al., 2023; Skerven et al., 2019). Clinicians need to sensitively address clients' experiences of minority stress and adjust DBT in consideration of the above recommendations.

In relation to structural health inequalities, the findings provide some support for the use of a GSRD‐centric framework to help realise the clinical implications for DBT mentioned in Table 3. Adopting the Intersectional, Social and Systems‐based framework (Dunlop & Lea, 2023a) could educate and support DBT therapists to ask about sexuality and experiences of homophobia and heterosexism within DBT pre‐treatment, skills group and during individual sessions. This could be especially helpful when doing behavioural chain analyses to formulate the sexuality‐specific internal and external factors implicated in self‐harm and suicidal behaviours, as well as possible areas to focus solution analyses and behavioural rehearsal on to reduce these in the future.

Overall, LGP face a difficult but rewarding journey throughout their DBT programmes. Many participants were able to experience a chosen family with other DBT participants. It was also clear that the influence of the DBT therapists was an integral part of LGP's DBT experience. At times participants' sexuality felt invisible and times where it was visible, and this led to participants recommending a GSRD affirming DBT. The study emphasises the need for LGP to have support from other LGP within their DBT programme, so they can build social support, which improves their experience of DBT. Furthermore, the study discusses changes to the DBT manual, programme, physical space and therapists that would lead to GSRD affirming DBT, which would make clients feel more comfortable to discuss sexuality‐related difficulties. Overall, the study suggests that DBT is beneficial to LGP and supports past research advocating for its use with GSRD individuals. This includes adapting DBT techniques to address sexuality‐related difficulties, fostering opportunities to create chosen families and contributing to creating a life worth living.

## AUTHOR CONTRIBUTIONS

**Charlotte Harding:** Writing – original draft; formal analysis. **Daniel Pratt:** Writing – review and editing; supervision. **James Lea:** Supervision.

## CONFLICT OF INTEREST STATEMENT

All authors declare no conflict of interest.

## Supporting information


Data S1.

## Data Availability

The data that supports the findings of this study are available on request from the corresponding author. The data is not publicly available due to privacy and/or ethical restrictions.

## Footnotes

^1^
The Diagnostic and Statistical Manual of Mental Disorders (5th ed., text rev.; DSM–5; American Psychiatric Association, 2022) utilises ‘Borderline Personality Disorder’, whereas the International Classification of Disease system (ICD‐10, World Health Organisation, 2016) utilises ‘Emotionally Unstable Personality Disorder’.

^2^
Heterosexism refers to discrimination and marginalisation of non‐heterosexual individuals in belief that heterosexuality is the default sexuality (Jun, 2024).

^3^
Diary cards are a central component element to DBT which assist with tracking mood, behaviour and use of DBT skills. They are designed to aid individuals in recognising patterns and maintaining DBT skills.

^4^
‘Wise Mind’ is a DBT term for when individuals are aware of their emotions and considering facts. It is a synthesis and balance between being emotional (emotion mind) and rational (rational mind).

^5^
Radical acceptance is a distress tolerance skill which involves the acceptance of perpetual emotions, thoughts, and unchangeable circumstances (Linehan, 2015).

## References

[papt12555-bib-0001] Allen, L. (2010). Queer(y)ing the straight researcher: The relationship(?) between researcher identity and anti‐normative knowledge. Feminism & Psychology, 20(2), 147–165.

[papt12555-bib-0002] American Psychiatric Association (Ed.). (2022). Diagnostic and statistical manual of mental disorders (5th ed.). American Psychiatric Association. 10.1176/appi.books.9780890425787

[papt12555-bib-0003] Barnicot, K. , Couldrey, L. , Sandhu, S. , & Priebe, S. (2015). Overcoming barriers to skills training in borderline personality disorder: A qualitative interview study. PLoS One, 10(10), e0140635. 10.1371/journal.pone.0140635 26465757 PMC4605586

[papt12555-bib-0004] Birt, L. , Scott, S. , Cavers, D. , Campbell, C. , & Walter, F. (2016). Member checking: A tool to enhance trustworthiness or merely a nod to validation? Qualitative Health Research, 26(13), 1802–1811. 10.1177/1049732316654870 27340178

[papt12555-bib-0005] Booth, T. (2018). Sounds of still voices: Issues in the use of narrative methods with people who have learning difficulties. In L. Barton (Ed.), Disability and society (pp. 237–255). Routledge.

[papt12555-bib-0006] British Psychological Society . (2019). Guidelines for psychologists working with gender, sexuality and relationship diversity. British Psychological Society.

[papt12555-bib-0007] Brocki, J. M. , & Wearden, A. J. (2006). A critical evaluation of the use of interpretative phenomenological analysis (IPA) in health psychology. Psychology & Health, 21(1), 87–108. 10.1080/14768320500230185

[papt12555-bib-0008] Brooks, A. B. , Herrmann, P. L. , & Andreas, S. (2021). The use of banter in psychotherapy: A systematic literature review. Counselling and Psychotherapy Research, 21(3), 570–586. 10.1002/capr.12361

[papt12555-bib-0009] Brooks, V. R. (1981). Minority stress and lesbian women. Lexington Books.

[papt12555-bib-0010] Buerger, W. M. , & Miller, A. L. (2022). Humor, irreverent communication, and DBT. In Creative CBT with youth: Clinical applications using humor, play, superheroes, and improvisation (pp. 25–41). Springer International Publishing.

[papt12555-bib-0011] Camp, J. (2023). Dialectical behaviour therapy for sexual minority populations. In J. Semlyen & P. Rohleder (Eds.), Sexual minorities and mental health: Current perspectives and new directions (pp. 271–302). Springer International Publishing. 10.1007/978-3-031-37438-8_12

[papt12555-bib-0012] Camp, J. , Durante, G. , Cooper, A. , Smith, P. , & Rimes, K. A. (2024). Clinical outcomes for sexual and gender minority adolescents in a dialectical behaviour therapy programme. Behavioural and Cognitive Psychotherapy, 52(4), 337–355. 10.1017/S135246582400016X 38586939 PMC7616180

[papt12555-bib-0013] Camp, J. , Morris, A. , Wilde, H. , Smith, P. , & Rimes, K. A. (2023). Gender‐ and sexuality‐minoritised adolescents in DBT: A reflexive thematic analysis of minority‐specific treatment targets and experience. Cognitive Behaviour Therapist, 16, 326. 10.1017/S1754470X23000326 PMC7615396 38125010

[papt12555-bib-0014] Camp, J. , Vitoratou, S. , & Rimes, K. A. (2020). LGBQ+ self‐acceptance and its relationship with minority stressors and mental health: A systematic literature review. Archives of Sexual Behavior, 49(7), 2353–2373. 10.1007/s10508-020-01755-2 32504233 PMC7497468

[papt12555-bib-0015] Ceatha, N. , Mayock, P. , Campbell, J. , Noone, C. , & Browne, K. (2019). The power of recognition: A qualitative study of social connectedness and wellbeing through LGBT sporting, creative and social groups in Ireland. International Journal of Environmental Research and Public Health, 16(19), 3636. 10.3390/ijerph16193636 31569733 PMC6801602

[papt12555-bib-0016] Chang, C. J. , Halvorson, M. A. , Lehavot, K. , Simpson, T. L. , & Harned, M. S. (2023). Sexual identity and race/ethnicity as predictors of treatment outcome and retention in dialectical behavior therapy. Journal of Consulting and Clinical Psychology, 91(10), 614–621. 10.1037/ccp0000826 37261739 PMC10526887

[papt12555-bib-0017] Clancy, M. (2013). Is reflexivity the key to minimising problems of interpretation in phenomenological research? Nurse Researcher, 20(6), 12–16. 10.7748/nr2013.07.20.6.12.e1209 23909106

[papt12555-bib-0018] Cohen, J. M. , Norona, J. C. , Yadavia, J. E. , & Borsari, B. (2021). Affirmative dialectical behavior therapy skills training with sexual minority veterans. Cognitive and Behavioral Practice, 28(1), 77–91. 10.1016/j.cbpra.2020.05.008

[papt12555-bib-0019] Cunningham, K. , Wolbert, R. , & Lillie, B. (2004). It's about me solving my problems: Clients' assessments of dialectical behavior therapy. Cognitive and Behavioral Practice, 11(2), 248–256. 10.1016/S1077-7229(04)80036-1

[papt12555-bib-0020] Davies, D. , & Neves, S. (2023). Gender, sex and relationship diversity therapy. In T. Hanley & L. Winter (Eds.), The SAGE handbook of counselling and psychotherapy (5th ed.). Sage.

[papt12555-bib-0021] DeCou, C. R. , Comtois, K. A. , & Landes, S. J. (2019). Dialectical behavior therapy is effective for the treatment of suicidal behavior: A meta‐analysis. Behavior Therapy, 50(1), 60–72. 30661567 10.1016/j.beth.2018.03.009

[papt12555-bib-0022] Detrie, P. M. , & Lease, S. H. (2007). The relation of social support, connectedness, and collective self‐esteem to the psychological well‐being of lesbian, gay, and bisexual youth. Journal of Homosexuality, 53(4), 173–199. 10.1080/00918360802103449 18689197

[papt12555-bib-0023] Diamond, L. M. , & Alley, J. (2022). Rethinking minority stress: A social safety perspective on the health effects of stigma in sexually‐diverse and gender‐diverse populations. Neuroscience and Biobehavioral Reviews, 138, 104720. 10.1016/j.neubiorev.2022.104720 35662651

[papt12555-bib-0024] Dunlop, B. J. , & Lea, J. (2023a). It's not just in my head: An intersectional, social and systems‐based framework in gender and sexuality diversity. Psychology and Psychotherapy: Theory, Research and Practice, 96(1), 1–15. 10.1111/papt.12438 PMC10099476 36351776

[papt12555-bib-0025] Dunlop, B. J. , & Lea, J. (2023b). Clinical formulation. In J. Semlyen & P. Rohleder (Eds.), Sexual minorities and mental health. Palgrave Macmillan. 10.1007/978-3-031-37438-8_8

[papt12555-bib-0026] Freeman‐Coppadge, D. J. , & Langroudi, K. F. (2021). Beyond LGBTQ‐affirmative therapy: Fostering growth and healing through intersectionality. In K. L. Nadal & M. R. Scharrón‐del Río (Eds.), Queer psychology: Intersectional perspectives (pp. 159–179). Springer International Publishing.

[papt12555-bib-0027] Garcia, J. , Vargas, N. , Clark, J. L. , Magaña Álvarez, M. , Nelons, D. A. , & Parker, R. G. (2020). Social isolation and connectedness as determinants of well‐being: Global evidence mapping focused on LGBTQ youth. Global Public Health, 15(4), 497–519. 10.1080/17441692.2019.1682028 31658001 PMC7093214

[papt12555-bib-0028] Gillespie, C. , Murphy, M. , Kells, M. , & Flynn, D. (2022). Individuals who report having benefitted from dialectical behaviour therapy (DBT): A qualitative exploration of processes and experiences at long‐term follow‐up. Borderline Personality Disorder and Emotion Dysregulation, 9(1), 1–14. 35227318 10.1186/s40479-022-00179-9 PMC8885141

[papt12555-bib-0029] Goldblatt, H. , Karnieli‐Miller, O. , & Neumann, M. (2011). Sharing qualitative research findings with participants: Study experiences of methodological and ethical dilemmas. Patient Education and Counseling, 82(3), 389–395. 10.1016/j.pec.2010.12.016 21257280

[papt12555-bib-0030] Guba, E. G. , & Lincoln, Y. (1989). Fourth generation evaluation. Sage.

[papt12555-bib-0031] Hale, E. D. , Treharne, G. J. , & Kitas, G. D. (2007). Qualitative methodologies I: Asking research questions with reflexive insight. Musculoskeletal Care, 5(3), 139–147. 10.1002/msc.109 17610235

[papt12555-bib-0032] Hendricks, M. L. , & Testa, R. J. (2012). A conceptual framework for clinical work with transgender and gender nonconforming clients: An adaptation of the minority stress model. Professional Psychology: Research and Practice, 43, 460–467. 10.1037/a0029597

[papt12555-bib-0033] Hodgetts, A. , Wright, J. , & Gough, A. (2007). Clients with borderline personality disorder: Exploring their experiences of dialectical behaviour therapy. Counselling and Psychotherapy Research, 7(3), 172–177. 10.1080/14733140701575036

[papt12555-bib-0034] Hudson, K. D. , & Bruce‐Miller, V. (2023). Nonclinical best practices for creating LGBTQ‐inclusive care environments: A scoping review of gray literature. Journal of Gay & Lesbian Social Services, 35(2), 218–240. 10.1080/10538720.2022.2057380

[papt12555-bib-0035] Jackman, K. , Honig, J. , & Bockting, W. (2016). Nonsuicidal self‐injury among lesbian, gay, bisexual and transgender populations: An integrative review. Journal of Clinical Nursing, 25(23–24), 3438–3453. 10.1111/jocn.13236 27272643

[papt12555-bib-0036] Jones, A. , Robinson, E. , Oginni, O. , Rahman, Q. , & Rimes, K. A. (2017). Anxiety disorders, gender nonconformity, bullying and self‐esteem in sexual minority adolescents: Prospective birth cohort study. Journal of Child Psychology and Psychiatry, and Allied Disciplines, 58(11), 1201–1209. 10.1111/jcpp.12757 28569044

[papt12555-bib-0037] Joyce, E. , Pratt, D. , & Lea, J. (2024). “Where is my place?” A qualitative study of gay men's experiences of social support, relationships and community in relation to psychological wellbeing and distress. Journal of Homosexuality, 1–27. 10.1080/00918369.2024.2354408 38787790

[papt12555-bib-0038] Jun, H. (2024). Heterosexism. In Social justice, multicultural counseling, and practice: Beyond a conventional approach (pp. 205–239). Springer Nature Switzerland.

[papt12555-bib-0039] Kayrouz, R. , Dear, B. F. , Karin, E. , & Titov, N. (2016). Facebook as an effective recruitment strategy for mental health research of hard to reach populations. Internet Interventions, 4, 1–10. 30135786 10.1016/j.invent.2016.01.001 PMC6096235

[papt12555-bib-0040] King, M. , Semlyen, J. , Tai, S. S. , Killaspy, H. , Osborn, D. , Popelyuk, D. , & Nazareth, I. (2008). A systematic review of mental disorder, suicide, and deliberate self harm in lesbian, gay and bisexual people. BMC Psychiatry, 8, 70. 10.1186/1471-244X-8-70 18706118 PMC2533652

[papt12555-bib-0041] Lakeman, R. , & Emeleus, M. (2020). The process of recovery and change in a dialectical behaviour therapy programme for youth. International Journal of Mental Health Nursing, 29(6), 1092–1100. 32535985 10.1111/inm.12749

[papt12555-bib-0042] Larkin, M. , Watts, S. , & Clifton, E. (2006). Giving voice and making sense in interpretative phenomenological analysis. Qualitative Research in Psychology, 3(2), 102–120.

[papt12555-bib-0043] Lea, J. , Jones, R. , & Huws, J. (2010). Gay psychologists and gay clients: Exploring therapist disclosure of sexuality in the therapeutic closet. Psychology of Sexualities Review, 1(1), 59–73. 10.53841/bpssex.2010.1.1.59

[papt12555-bib-0044] Linehan, M. M. (1993). Cognitive behavioral treatment of borderline personality disorder. Guilford Press.

[papt12555-bib-0045] Linehan, M. M. (2015). DBT skills training manual. The Guilford Press.

[papt12555-bib-0046] Little, H. , Tickle, A. , & das Nair, R. (2018). Process and impact of dialectical behaviour therapy: A systematic review of perceptions of clients with a diagnosis of borderline personality disorder. Psychology and Psychotherapy: Theory, Research and Practice, 91(3), 278–301. 10.1111/papt.12156 29034599

[papt12555-bib-0047] McClain, Z. , Hawkins, L. A. , & Yehia, B. R. (2016). Creating welcoming spaces for lesbian, gay, bisexual, and transgender (LGBT) patients: An evaluation of the health care environment. Journal of Homosexuality, 63(3), 387–393. 10.1080/00918369.2016.1124694 26643126

[papt12555-bib-0048] McNeill, S. G. , McAteer, J. , & Jepson, R. (2023). Interactions between health professionals and lesbian, gay and bisexual patients in healthcare settings: A systematic review. Journal of Homosexuality, 70(2), 250–276. 10.1080/00918369.2021.1945338 34292130

[papt12555-bib-0049] Meyer, I. H. (1995). Minority stress and mental health in gay men. Journal of Health and Social Behavior, 36(1), 38–56. 7738327

[papt12555-bib-0050] Meyer, I. H. (2003). Prejudice, social stress, and mental health in lesbian, gay, and bisexual populations: Conceptual issues and research evidence. Psychological Bulletin, 129(5), 674–697. 10.1037/0033-2909.129.5.674 12956539 PMC2072932

[papt12555-bib-0051] Meyer, I. H. (2010). Identity, stress, and resilience in lesbians, gay men, and bisexuals of color. The Counseling Psychologist, 38(3), 442–454. 10.1177/0011000009351601 PMC3860594 24347674

[papt12555-bib-0052] Meyer, I. H. (2015). Resilience in the study of minority stress and health of sexual and gender minorities. Psychology of Sexual Orientation and Gender Diversity, 2(3), 209–213.

[papt12555-bib-0053] Miga, E. M. , Neacsiu, A. D. , Heard, H. L. , & Dimeff, L. A. (2019). Dialectical behaviour therapy from 1991–2015: What do we know about clinical efficacy and research quality? In M. Swales (Ed.), Oxford handbook of DBT (pp. 415–465). OUP.

[papt12555-bib-0054] Moagi, M. M. , van Der Wath, A. E. , Jiyane, P. M. , & Rikhotso, R. S. (2021). Mental health challenges of lesbian, gay, bisexual and transgender people: An integrated literature review. Health SA = SA Gesondheid, 26, 1487. 10.4102/hsag.v26i0.1487 33604059 PMC7876969

[papt12555-bib-0055] Office for National Statistics . (2023, August 31). Inequalities in mortality involving common physical health conditions, England: 21 march 2021 to 31 January 2023. ONS. https://www.ons.gov.uk/peoplepopulationandcommunity/healthandsocialcare/healthinequalities/bulletins/inequalitiesinmortalityinvolvingcommonphysicalhealthconditionsengland/21march2021to31january2023

[papt12555-bib-0056] Ohlis, A. , Bjureberg, J. , Ojala, O. , Kerj, E. , Hallek, C. , Fruzzetti, A. E. , & Hellner, C. (2023). Experiences of dialectical behaviour therapy for adolescents: A qualitative analysis. Psychology and Psychotherapy: Theory, Research and Practice, 96(2), 410–425. 10.1111/papt.12447 36756991

[papt12555-bib-0057] O'Shaughnessy, T. , & Speir, Z. (2018). The state of LGBQ affirmative therapy clinical research: A mixed‐methods systematic synthesis. Psychology of Sexual Orientation and Gender Diversity, 5(1), 82–98. 10.1037/sgd0000259

[papt12555-bib-0058] Oshin, L. A. , Silamongkol, T. , Pucker, H. , Finkelstein, J. , King, A. M. , & Rizvi, S. L. (2024). … And we could do better: Comparing the efficacy of dialectical behavior therapy between LGBQ and heterosexual adults with borderline personality disorder. Professional Psychology: Research and Practice, 55(1), 58–67. 10.1037/pro0000529

[papt12555-bib-0059] Oud, M. , Arntz, A. , Hermens, M. L. M. , Verhoef, R. , & Kendall, T. (2018). Specialized psychotherapies for adults with borderline personality disorder: A systematic review and meta‐analysis. Australian and New Zealand Journal of Psychiatry, 52(10), 949–961. 30091375 10.1177/0004867418791257 PMC6151959

[papt12555-bib-0060] Panos, P. T. , Jackson, J. W. , Hasan, O. , & Panos, A. (2014). Meta‐analysis and systematic review assessing the efficacy of dialectical behavior therapy (DBT). Research on Social Work Practice, 24(2), 213–223. 10.1177/1049731513503047 30853773 PMC6405261

[papt12555-bib-0061] Pantalone, D. W. , Sloan, C. A. , & Carmel, A. (2019). Dialectical behavior therapy for borderline personality disorder and suicidality among sexual and gender minority individuals. In J. E. Pachankis & S. A. Safren (Eds.), Handbook of evidence‐based mental health practice with sexual and gender minorities (pp. 408–429). Oxford University Press.

[papt12555-bib-0062] Pinto‐Coelho, K. G. , Hill, C. E. , & Kivlighan, D. M., Jr. (2016). Therapist self‐disclosure in psychodynamic psychotherapy: A mixed methods investigation. Counselling Psychology Quarterly, 29(1), 29–52. 10.1080/09515070.2015.1072496

[papt12555-bib-0063] Poon, J. , Galione, J. N. , Grocott, L. R. , Horowitz, K. J. , Kudinova, A. Y. , & Kim, K. L. (2022). Dialectical behavior therapy for adolescents (DBT‐A): Outcomes among sexual minorities at high risk for suicide. Suicide & Life‐Threatening Behavior, 52(3), 383–391. 10.1111/sltb.12828 35019159 PMC9233065

[papt12555-bib-0064] Porter, J. , Hulbert‐Williams, L. , & Chadwick, D. (2015). Sexuality in the therapeutic relationship: An interpretative phenomenological analysis of the experiences of gay therapists. Journal of Gay & Lesbian Mental Health, 19(2), 165–183. 10.1080/19359705.2014.957882

[papt12555-bib-0065] Rathus, J. H. , & Miller, A. L. (2015). DBT® skills manual for adolescents. Guilford Press.

[papt12555-bib-0066] Schlichter, A. (2004). Queer at last? Straight intellectuals and the desire for transgression. GLQ: A Journal of Lesbian and Gay Studies, 10(4), 543–564.

[papt12555-bib-0067] Semlyen, J. , King, M. , Varney, J. , & Hagger‐Johnson, G. (2016). Sexual orientation and symptoms of common mental disorder or low wellbeing: Combined meta‐analysis of 12 UK population health surveys. BMC Psychiatry, 16, 67. 10.1186/s12888-016-0767-z 27009565 PMC4806482

[papt12555-bib-0068] Skerven, K. , Whicker, D. , & LeMaire, K. (2019). Applying dialectical behaviour therapy to structural and internalized stigma with LGBTQ clients. The Cognitive Behaviour Therapist, 12, E9. 10.1017/S1754470X18000235

[papt12555-bib-0201] Skerven, K. , Mirabito, L. , Kirkman, M. , & Shaw, B. (2021). Dialectical behaviour therapy skills group including stigma management: A pilot with sexual and gender minority veterans. The Cognitive Behaviour Therapist, 14, E33. 10.1017/S1754470X21000295

[papt12555-bib-0069] Smith, J. A. , Flowers, P. , & Larkin, M. (2009). Analysis: Theory, method and research. Sage.

[papt12555-bib-0070] Smith, J. A. , & Osborn, M. (2003). Interpretative phenomenological analysis. In J. A. Smith (Ed.), Qualitative psychology: A practical guide to research methods (pp. 53–80). Sage Publications Ltd.

[papt12555-bib-0071] Smith, J. A. , & Osborn, M. (2007). Pain as an assault on the self: An interpretative phenomenological analysis of the psychological impact of chronic benign low back pain. Psychology and Health, 22(5), 517–534.

[papt12555-bib-0072] Spengler, E. S. , Miller, D. J. , & Spengler, P. M. (2016). Microaggressions: Clinical errors with sexual minority clients. Psychotherapy, 53(3), 360–366. 10.1037/pst0000073 27631867

[papt12555-bib-0073] Stonewall . (2018). LGBT in Britain – Health report . Stonewall. https://www.stonewall.org.uk/system/files/lgbt_in_britain_health.pdf

[papt12555-bib-0074] Swales, M. (2020). Reducing suicidal behaviour in people with complex needs: Addressing the challenge of implementing dialectical behaviour therapy. Suicidologi, 24(3), 4–14. 10.5617/suicidologi.7689

[papt12555-bib-0075] Swannell, S. , Martin, G. , & Page, A. (2016). Suicidal ideation, suicide attempts and non‐suicidal self‐injury among lesbian, gay, bisexual and heterosexual adults: Findings from an Australian national study. The Australian and New Zealand Journal of Psychiatry, 50(2), 145–153. 10.1177/0004867415615949 26631718

[papt12555-bib-0076] Teh, Y. Y. , & Lek, E. (2018). Culture and reflexivity: Systemic journeys with a British Chinese family. Journal of Family Therapy, 40(4), 520–536. 10.1111/1467-6427.12205

[papt12555-bib-0077] Thomas, C. (Ed.). (2000). Straight with a twist: Queer theory and the subject of heterosexuality. University of Illinois Press.

[papt12555-bib-0078] Turpin, G. , Barley, V. , Beail, N. , Scaife, J. , Slade, P. , Smith, J. , & Walsh, S. (1997). Standards for research projects and theses involving qualitative methods: Suggested guidelines for trainees and courses. Clinical Psychology Forum, 108, 3–7.

[papt12555-bib-0079] Woodhead, C. , Gazard, B. , Hotopf, M. , Rahman, Q. , Rimes, K. A. , & Hatch, S. L. (2016). Mental health among UK inner city non‐heterosexuals: The role of risk factors, protective factors and place. Epidemiology and Psychiatric Sciences, 25(5), 450–461. 10.1017/S2045796015000645 26264675 PMC7137594

[papt12555-bib-0080] World Health Organization . (2016). International statistical classification of diseases and related health problems (10th ed.). WHO. https://icd.who.int/browse10/2016/en 10.2471/BLT.25.294650 PMC13531092 42683092

