# Supplementary material for: “All the horrible emotions have passed, I still remained, and I was safe”: A qualitative study of Lesbian and Gay people's lived experience of completing a full Dialectical Behaviour Therapy programme
Source: Psychol Psychother. 2024 Oct 30;98(1):1–24. doi: 10.1111/papt.12555 (PMC11823363; doi:10.1111/papt.12555)
Supplement: Supplementary file 1 — Data S1. [file PAPT-98-1-s001.docx]

**Appendix**

Appendix A

| **Sample of study topic guide** | |
| --- | --- |
| **Section 1: Life before you had DBT**  As a gay or lesbian person, can you tell me about your difficulties that brought you into DBT? | *Can you tell me a little bit more about life before starting treatment as a gay or lesbian person?*  *Can you tell me a little bit more about your past suicidal thoughts?*  *Was their anyone in your life, prior to DBT, who was not supportive of your sexuality?* |
| **Section 2: Experience of DBT**  As a lesbian or gay person, can you tell me about your experience of going through and completing Dialectical Behavioural Therapy (DBT)? | *What parts were helpful/unhelpful?*  *How did you find engaging with DBT whilst possibly experiencing self-harming and/or suicidal thoughts?*  *How did you find the DBT therapists?* |
| **Section 3: Engaging in DBT as a minoritized sexuality**  As a gay or lesbian person, can you tell me about how you found engaging with DBT as a person with a marginalised identity? | *How did you feel being gay/lesbian in DBT?*  *Can you tell me about how DBT addressed the invalidating environment you experienced due to identifying as a gay or lesbian adult?*  *Did anyone ever mention minority stress and how to relates to the invalidating environment?* |
| **Section 4**  Do you think DBT was affirmative to you as a gay or lesbian person? | *Did you feel your sexuality was accepted in DBT?*  *Did your DBT therapist acknowledge the challenges and difficulties you face being a lesbian or gay person?*  *Are there any changes you would make to the individual therapy or group modules to make DBT more affirmative?* |
